# PrimPol—Prime Time to Reprime

**DOI:** 10.3390/genes8010020

**Published:** 2017-01-06

**Authors:** Thomas A. Guilliam, Aidan J. Doherty

**Affiliations:** Genome Damage and Stability Centre, School of Life Sciences, University of Sussex, Brighton BN1 9RQ, UK; T.Guilliam@sussex.ac.uk

**Keywords:** primase, polymerase, AEP, PrimPol, DNA replication, priming, translesion synthesis, damage tolerance

## Abstract

The complex molecular machines responsible for genome replication encounter many obstacles during their progression along DNA. Tolerance of these obstructions is critical for efficient and timely genome duplication. In recent years, primase-polymerase (PrimPol) has emerged as a new player involved in maintaining eukaryotic replication fork progression. This versatile replicative enzyme, a member of the archaeo-eukaryotic primase (AEP) superfamily, has the capacity to perform a range of template-dependent and independent synthesis activities. Here, we discuss the emerging roles of PrimPol as a leading strand repriming enzyme and describe the mechanisms responsible for recruiting and regulating the enzyme during this process. This review provides an overview and update of the current PrimPol literature, as well as highlighting unanswered questions and potential future avenues of investigation.

## 1. Introduction

The eukaryotic replisome is a highly co-ordinated complex of molecular machines, tasked with efficiently duplicating the genome whilst maintaining a near-perfect level of accuracy. At the heart of the replisome lie the classical DNA polymerases (Pols) α, δ, and ε, which together perform the bulk of the “reading” and copying during replication [1]. These enzymes are exceptionally specialised to faithfully duplicate intact DNA but, consequently, are also highly sensitive to perturbations in the DNA template, resulting in the slowing and stalling of replication forks in the presence of replication stress [2]. 

Many endogenous and exogenous sources contribute to replication stress. Unrepaired DNA lesions generated by inherent metabolic processes within the cell, in addition to external chemical and physical mutagens, serve as potent blocks to the progression of the canonical replicative DNA Pols [3]. Furthermore, non-B DNA secondary structures and collisions between the replisome and transcription machinery also lead to fork stalling and potentially collapse [4,5]. Aside from direct blockages, repetitive DNA sequences, common fragile sites, ribonucleotides incorporated in the template strand, and limiting pools of nucleotides, can all also act as sources of replication stress [6,7,8]. 

In order to maintain replication in the presence of stalled and damaged replication forks, eukaryotes possess a number of distinct damage tolerance and fork restart mechanisms [9]. The deployment of these mechanisms differs depending on which template strand is affected. Obstacles encountered on the lagging strand are easily overcome due to the discontinuous nature of lagging strand replication, where primers are repeatedly generated for Okazaki fragment synthesis. This allows resumption of lagging strand synthesis downstream of a lesion, or obstacle, through use of a newly generated primer to reinitiate replication, leaving behind a single-stranded DNA (ssDNA) gap [10,11]. 

The situation on the leading strand is more complex with numerous restart pathways available [9]. However, in the last decade evidence has emerged indicating that repriming downstream of lesions and secondary structures also occurs on the leading strand, suggesting that eukaryotic leading strand replication is not exclusively continuous as first thought [11,12]. Indeed, repriming of leading strand replication in prokaryotes is now well documented [13,14]. In addition to repriming, stalled replication forks can also utilise translesion synthesis (TLS) to directly replicate over damaged nucleobases in the template strand. Here, specialised, but error-prone, damage tolerant Pols, predominantly of the Y-family, replace the replicative Pol and synthesise a short section of DNA over the lesion, before handing back over to the replicase [15,16]. It is widely believed that TLS can occur both at the replication fork and post-replicatively to fill in ssDNA gaps left opposite damaged bases as a result of repriming [17,18,19,20]. Such gaps may also appear due to the firing of dormant replication origins downstream of a stalled fork, a process which can itself rescue replication [21]. Aside from TLS, ssDNA gaps left opposite lesions can also be filled in an error-free manner through recombination-mediated template switching. Here, the newly synthesised undamaged sister chromatid is used as a template for extension [22]. Template switching may additionally occur at the replication fork via fork reversal; remodelling of the stalled fork generates a four-way junction through annealing of the two nascent DNA strands, thus providing an undamaged template for continued extension [23]. 

DNA damage tolerance, in particular TLS, has long been associated with the specialised damage tolerant Pols of the Y-family. However, more recently it is becoming clear that members of the archaeo-eukaryotic primase (AEP) superfamily also display novel roles in DNA damage tolerance and repair pathways [24]. In archaea, where many species lack Y-family TLS Pols, the replicative primase is inherently TLS proficient [25]. This review will focus on a new player in eukaryotic nuclear and mitochondrial DNA damage tolerance, and only the second human AEP to be identified, primase-polymerase (PrimPol) (alternative names CCDC111, FLJ33167, EukPrim2 or PrimPol1), encoded by the *PRIMPOL* gene on chromosome 4q35.1 [26,27,28]. After an overview and evolutionary history, we will describe the domain architecture and biochemical features of the enzyme before moving on to discuss recent advances in our understanding of its roles, recruitment and regulation in vertebrate cells. 

## 2. Discovery and Evolutionary History of PrimPol

The AEP superfamily is evolutionarily and structurally distinct from the bacterial DnaG-type primases which, like AEPs in archaea and eukarya, are absolutely required for DNA replication initiation in bacteria [24]. Nevertheless, DnaG-like primases are also present in archaea, and likewise, AEPs have been identified in bacteria [29]. In each case, these enzymes have diverged to fulfil alternative roles, for example in bacteria a member of the AEP superfamily is employed, together with Ku and DNA ligase homologues, in a non homologous end-joining (NHEJ) DNA break repair pathway [30,31]. It is likely that the presence of AEPs in bacteria is a result of horizontal gene transfer (HGT), with the enzymes originally being recruited for replication initiation by the archaeo-eukaryotic lineage following their divergence from bacteria [24,32]. The catalytic core of AEPs is defined by two structural modules; an N-terminal module with an (αβ)_2_ unit, and a C-terminal RNA recognition module-like (RRM-like) fold. These two modules pack together, with the active site residues located in between them [32]. 

In 2005, detailed *in silico* analyses divided the AEP superfamily into 13 major families, which were further organised into three higher order clades; the AEP proper clade, the nucleo-cytoplasmic large DNA virus (NCLDV)-herpesvirus primase clade, and the primpol clade [32]. These analyses also identified PrimPol and assigned it to the NCLDV-herpesvirus clade, whose members are only present in eukaryotes and their viruses. This clade encompasses the iridovirus primase and herpes-pox primase families, PrimPol belonging to the latter. Members of the herpes-pox primase family possess a conserved C-terminal β-strand-rich region, which replaces the Primase C Terminal (PriCT) domain of the iridovirus primase family [32]. The NCLDV-herpesvirus primase clade is suggested to have originated from bacteriophage or bacterial proteins possessing a fused AEP and PriCT-2 domain. Herpes viruses likely acquired their primase from the NCLDV class, before replacing the C-terminal PriCT domain with the characteristic β-strand-rich region [32]. 

PrimPol orthologues are conserved across vertebrates, plants, and primitive eukaryotes including species of fungi, algae, and protists, such as apicomplexans and the slime mold *Dictyostelium*. However, PrimPol is notably absent from prokaryotes and a number of fungi and animal species, including *Caenorhabditis elegans* and *Drosophila* [26,27,32]. This interrupted distribution of PrimPol, coupled with the diversity of AEPs observed in mobile elements such as viruses and plasmids [24], suggests that PrimPol was originally obtained through HGT by an early eukaryote and then lost on multiple separate occasions. Importantly, PrimPol is not closely related to the eukaryotic replicative DNA primase small subunit (Prim1), a member of the AEP-proper clade, and is dispensable for DNA replication in higher eukaryotes [26,32]. It has been speculated that PrimPol may have originated as a DNA repair enzyme in NCLDVs, potentially required due to their large genome size and lack of access to cellular DNA repair enzymes during replication [32]. Likewise, PrimPol may play a role in DNA replication initiation in these viruses. 

## 3. What Can PrimPol Do? The Domain Architecture and Catalytic Activities of PrimPol

**Figure 1:**
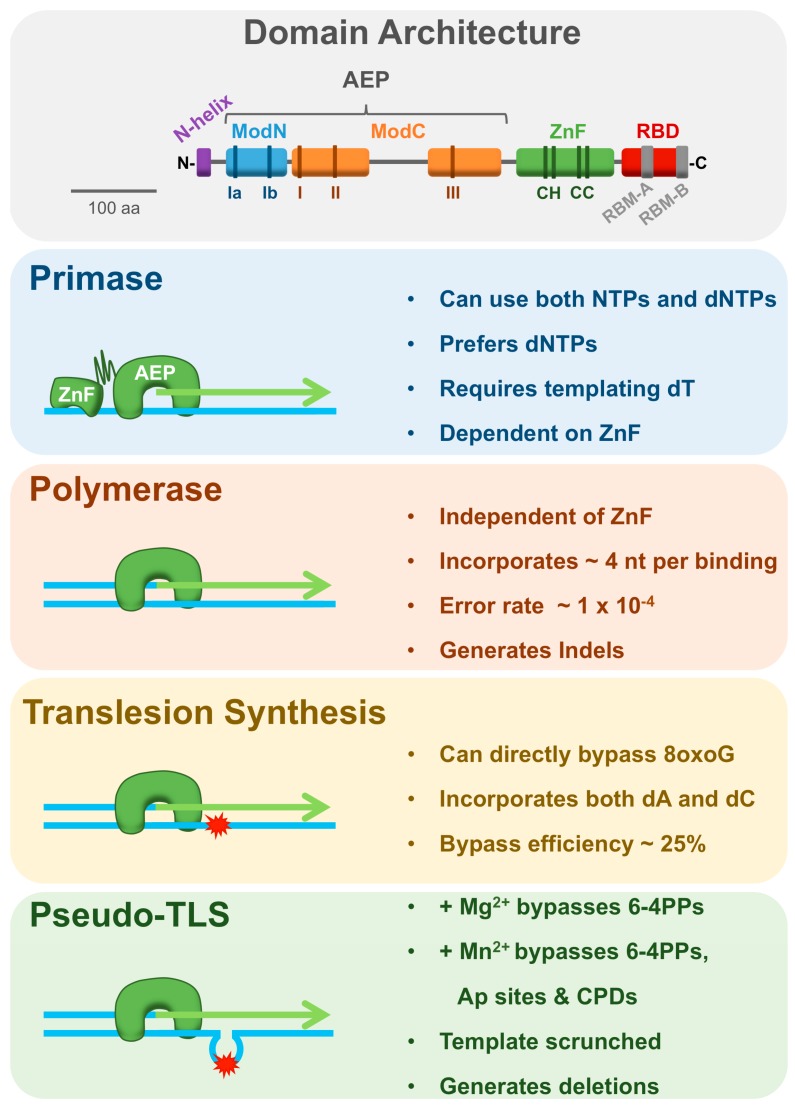
Domain architecture and catalytic activities of primase-polymerase (PrimPol). The domain architecture of PrimPol is depicted in the top panel. A helix (**purple**) located at the N-terminus is connected to ModN by a flexible linker and contacts the DNA major groove. ModN (**blue**) and ModC (**orange**) comprise the archaeo-eukaryotic primase (AEP) domain and contain motifs Ia, Ib, I, II, and III, required for template binding and catalytic activity. The zinc finger (ZnF) (**green**) contains three conserved cysteines and a histidine which coordinate a zinc ion and are required for primase, but not polymerase, activity. The replication protein A (RPA) binding domain (RBD) (**red**) containing RPA binding motif-A (RBM-A) and RBM-B (**grey**) is located at the C-terminus. A 100 amino acid (aa) scale bar is shown to the right. The catalytic activities of PrimPol are displayed below.

Since the initial identification of PrimPol in 2005 [32], a number of groups have purified and characterised the recombinant protein, permitting insight into the architectural and biochemical properties of the enzyme [26,27,28]. These studies revealed PrimPol’s impressive range of nucleotidyl transferase activities, suggesting a number of potential roles in vivo. In this section, we will describe these activities and the domain architecture of the protein, which underpins its catalytic flexibility (Figure 1).

### 3.1. Domain Architecture and Structure

Previously, an alignment of PrimPol homologues identified 14 conserved regions within the protein, including three characteristic AEP catalytic motifs (motifs I, II, and III) towards the N-terminus, forming the AEP domain [32]. Interestingly, motif I displays the variant DxE, rather than the typical DxD motif possessed by most AEPs. Motif I and motif III (xD) together form the divalent metal ion binding site and are essential for the catalytic activity of the enzyme. Motif II (SxH) was predicted to form part of the nucleotide binding site, and is again required for all catalytic activity [26,27,33,34]. Recently, the crystal structure of a ternary complex of the AEP domain of PrimPol (residues 1–354) bound to a DNA template-primer and incoming nucleotide was elucidated, confirming the existence and role of these motifs and two additional motifs, Ia (RQ) and Ib (QRhY/F), which interact with the template DNA strand [35]. The structure reveals that PrimPol’s catalytic core encloses the 3’-end of the primer with two α/β modules, ModN and ModC, lining the cavity. ModN primarily interacts with the template strand, whilst ModC contains the catalytic residues and interacts with the incoming nucleotide, as well as the template strand. Intriguingly, the structure of PrimPol’s AEP domain does not resemble a typical polymerase fold in any way. There is no thumb domain to hold the primer-template, in fact the primer DNA strand almost completely lacks protein contacts, and ModC was shown to function as both the finger and palm domains [35]. 

PrimPol also possesses a second conserved domain, a C-terminal UL52-like zinc finger (ZnF) containing three conserved cysteines and a histidine, as is typical for herpes-pox primase family members [32]. The first conserved cysteine and histidine residues of this domain coordinate a zinc ion and are critical for the primase, but not polymerase, activity of the enzyme [34,36]. 

### 3.2. Primase Activity

As predicted by the initial *in silico* identification, PrimPol is an active primase that is able to utilise both NTPs and dNTPs for primer synthesis, a unique ability amongst eukaryotic enzymes [26,27,28]. Surprisingly, PrimPol actually displays a preference for dNTPs over NTPs during primer synthesis, a feature more typically associated with archaeal primases [26]. Similar to the requirement of templated pyrimidines for dinucleotide synthesis by Prim1, PrimPol only generates primers on dT containing templates [26,37]. This primase activity is dependent upon an intact ZnF domain, which is consistent with previous studies on the herpes simplex virus type I (HSV1) helicase/primase complex. Here, primase activity was lost when key residues in the UL52 zinc-binding domain were mutated [38,39]. Interestingly, the ZnF domain of PrimPol has been shown to bind single-stranded (ss) but not double-stranded (ds) DNA, suggesting that this module may be important for stabilising PrimPol on ssDNA templates to allow synthesis of the initial dinucleotide [34]. The ability of PrimPol to synthesise DNA primers *de novo* gives it the potential to reprime and restart replication downstream of DNA damage lesions and fork-stalling obstacles in vivo.

### 3.3. Polymerase and Lesion Bypass Activities

In addition to its DNA and RNA primase activity, PrimPol is also a template-dependent DNA polymerase, with an ability to bypass a number of DNA damage lesions. Notably, PrimPol can bypass both oxidative and ultraviolet (UV)-induced lesions, including 8-oxo-guanine (8oxoG), and pyrimidine (6-4) pyrimidone photoproducts (6-4PPs) [26,27]. A recent study analysing the kinetics of 8oxoG bypass by PrimPol found that the enzyme incorporates dC (error free) opposite the lesion with 6-fold higher efficiency than dA (error prone). Incorporation of dC opposite 8oxoG occurred at ≈25% efficiency compared to an unmodified templating dG, suggesting that PrimPol has the potential to function as an efficient TLS Pol in vivo [40]. However, the accuracy of bypass differs in other reports, in some instances being only 50% error-free [26,34,41,42]. In the case of 6-4PPs, PrimPol bypasses the lesion in an error-prone manner [26]. Although unable to directly traverse a cyclobutane pyrimidine dimer (CPD), PrimPol can extend from mismatched bases opposite a CPD [26]. Additionally, a truncated form of PrimPol, lacking the ZnF domain, can facilitate TLS past a CPD [34]. In contrast, in the presence of manganese, PrimPol’s TLS activity is altered allowing the full-length enzyme to extend past cyclobutane pyrimidine dimers (CPDs) and abasic sites (Ap sites), in addition to 6-4PPs and 8oxoG lesions [36]. However, the usage of either magnesium or manganese as the primary cofactor for PrimPol in vivo remains unclear. 

### 3.4. Lesion Skipping and Template Independent Extension

Despite displaying the ability to directly read through some damaged nucleobases, such as 8oxoG, it appears that PrimPol’s bypass of more bulky or distorting lesions is facilitated through a pseudo-TLS mechanism. Here, PrimPol is able to re-anneal the primer to a new position downstream of the lesion prior to extension, thus looping out the templating lesion and generating a shorter extension product than would be produced from strict template-dependent extension [27,36,43]. This activity is enhanced in the presence of manganese, permitting bypass of 6-4PPs, CPDs, and Ap sites by pseudo-TLS [27,36]. Intriguingly, this characteristic is reminiscent of the Ap site bypass strategy employed by the primase/polymerisation domain (PolDom) of *Mycobacterium tuberculosis* DNA ligase (LigD) [44,45]. The ability of manganese to stimulate primer-realignment and template scrunching by PrimPol offers a clear explanation for the altered TLS ability of the enzyme in the presence of this metal ion. It has also been reported that manganese increases both PrimPol’s polymerase activity and affinity for DNA, compared to magnesium [40]. 

Notably however, manganese also promotes promiscuous template-independent extension by PrimPol, resulting in the generation of non-complementary homopolymeric strands [34]. The mutagenic effect of manganese on polymerase activity, through increased reactivity and promotion of non-template-directed nucleotidyl transfer, has been clear for several decades [46,47,48,49,50]. Moreover, the bypass of lesions via template scrunching is potentially more detrimental than beneficial to genomic integrity, due to the high risk of generating frame-shift mutations. Therefore, it seems likely that more low-risk mechanisms would be employed in vivo where available. 

The lower affinity of PrimPol for DNA and incoming nucleotides in the presence of magnesium is often taken as support for manganese as the enzyme’s primary metal ion cofactor in vivo [40]. However, PrimPol’s inherent low affinity for DNA and dNTPs, when using magnesium as a cofactor, may actually act as an important mechanism to regulate its activity. In support of this, it has previously been shown that dNTP levels in yeast are increased 6–8-fold in the presence of DNA damage [51]. Importantly, TLS Pols often require ≈10 times greater dNTP concentrations for nucleotide binding opposite a lesion, compared to a replicative Pol at an undamaged site [52,53]. Increased intracellular dNTP concentrations have been found to correlate with an increase in damage tolerance, but also increased mutation rates, potentially due to the unregulated participation of TLS Pols in ‘normal’ replication [51]. Thus, in yeast it appears that the in vivo activity of TLS Pols is partly regulated by dNTP levels, which increase after DNA damage, consequently restricting the contribution of these Pols to ‘normal’ DNA replication. Intriguingly, ribonucleotide reductase has been found to be up-regulated in response to DNA damage in all studied organisms, suggesting that increased dNTP synthesis in response to damage may be a conserved mechanism across all domains of life [54]. Similarly, PrimPol’s relatively poor affinity for DNA may be overcome in vivo by association with other factors, such as replication protein A (RPA) and polymerase-delta interacting protein 2 (PolDIP2 or PDIP38), again acting to regulate the enzyme by only recruiting it to loci where it is actually required [28,41,55]. 

Additionally, it is not clear whether the relatively low intracellular concentrations of manganese (0.1 to 40 µM) [56,57,58], compared to magnesium (0.21 to 0.24 mM) [59,60], are sufficient to support the manganese-dependent TLS activities of PrimPol in vivo. Indeed, PrimPol required manganese concentrations of 200–1000 µM to facilitate pseudo-TLS bypass of an abasic site in vitro, whilst 100 µM did not permit any observable bypass [43]. Thus, the cellular relevance of these activities is not immediately clear. One intriguing possibility is that PrimPol utilises manganese in the mitochondria only [40]. Here, dNTP concentrations are lower than those in the cytosol, there is a dearth of TLS Pols, manganese uptake is increased in response to oxidative stress, and the high copy number nature of mitochondrial DNA (mtDNA) may allow more promiscuous lesion bypass mechanisms to be employed [40,61]. That said, more recent in vitro reconstitution experiments argue against a TLS-like role for PrimPol in oxidative damage bypass during mitochondrial DNA replication [42]. 

### 3.5. Fidelity, Mutagenic Signature, and Processivity

Typically, the price paid by Pols for DNA damage tolerance is a significant decrease in both fidelity and processivity. Whilst the structural features of replicative Pols confer extremely efficient and high fidelity DNA synthesis, TLS Pols possess more spacious active sites, altered finger and thumb domains, and lack proofreading exonuclease capabilities. These characteristics permit bypass of bulky lesions, but result in greatly decreased fidelity and processivity on undamaged DNA templates [16]. Likewise, the eukaryotic replicative primase exhibits poor fidelity compared to replicative Pols [62,63,64]. Rather unsurprisingly, PrimPol, which combines both TLS and primase capabilities, exhibits high error rates of ≈1 × 10^−4^, comparable with Y and X-family Pols [55]. Unlike these Pols however, PrimPol generates insertion-deletion (indel) errors at a much higher frequency than substitution mutations, which may be a result of its template scrunching ability [55]. Manganese acts to further decrease PrimPol’s fidelity on undamaged DNA and even more so on 8oxoG containing templates [40]. In addition to poor fidelity, PrimPol shares the characteristic of low processivity with canonical TLS Pols, incorporating only 1–4 nucleotides per binding event [34]. Intriguingly, the enzyme’s processivity was found to be negatively regulated by its ZnF domain, which may act to stabilise DNA binding and allow primer synthesis, whilst additionally limiting primer extension. Removal of the ZnF domain has also been found to lower PrimPol’s fidelity, suggesting the domain acts to regulate processivity and fidelity, as well as enabling primase activity [34]. 

## 4. What Does PrimPol Do? The Role of PrimPol in DNA Replication

**Figure 2:**
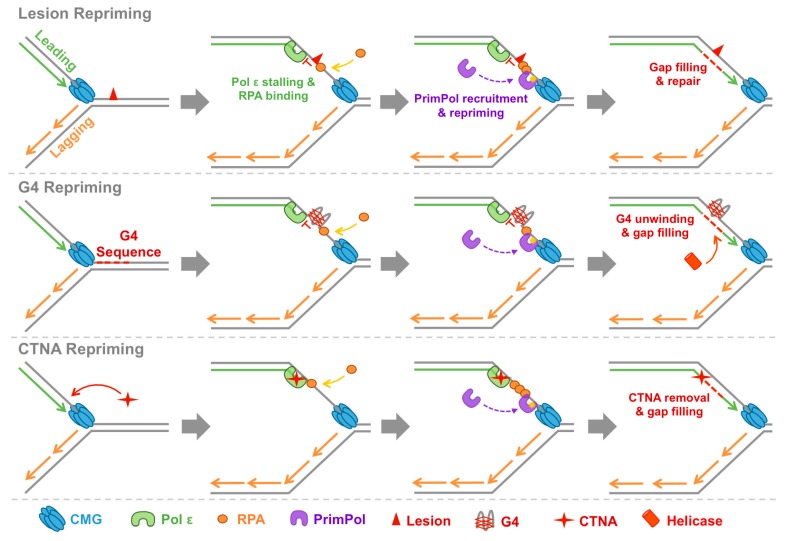
Repriming roles of PrimPol in nuclear DNA replication. PrimPol is able to reprime and reinitiate leading strand replication downstream of a range of replicase stalling obstacles. Here, the ability of PrimPol to reprime downstream of DNA lesions, G4 secondary structures, and chain-terminating nucleotide analogues, is highlighted. Following repriming, replication can proceed and the resulting single-stranded DNA (ssDNA) gap is filled through translesion synthesis (TLS) or template switching mechanisms, permitting subsequent repair or removal of the obstacle. Only the CDC45, MCM, GINS (CMG) complex, Pol ε, PrimPol, and RPA, are shown for simplicity. A key for identifying each factor is shown below. CTNA: chain-terminating nucleoside analogues.

The biochemical classification of PrimPol as both a RNA/DNA primase and a TLS Pol clearly suggests a role in DNA replication and damage tolerance. Moreover, these two characteristics give PrimPol the potential to assist the replisome in two different ways; through TLS or repriming. In this section, we will describe the in vivo characterisation of the enzyme, as well as the consequences of its deletion on the cell. Using this information, we will discuss recent advances in our understanding of the cellular roles of PrimPol (Figure 2). 

### 4.1. PrimPol—A DNA Damage Tolerance Enzyme

DNA damage tolerance is critical to support continued replisome progression in the presence of unrepaired DNA damage. An inability to tolerate this damage can lead to prolonged fork stalling, collapse and, ultimately, genome instability and/or cell death. The importance of DNA damage tolerance in preserving genomic integrity is highlighted by the consequences on human health of dysfunction in these mechanisms. An obvious example is the variant form of *xeroderma pigmentosum* (XPV). Here, mutation of Pol η, one of many Y-family TLS Pols, causes increased sensitivity to sunlight and a predisposition to skin cancer [16]. This is thought to occur due to mutagenic bypass of UV-induced CPDs by alternative TLS Pols. 

Interestingly, loss of PrimPol in human XPV cells leads to a synergistic increase in UV sensitivity, with the enzyme performing a distinct role from Pol η during this process [26]. In line with this, PrimPol forms sub-nuclear foci, and is recruited to chromatin, in response to UV irradiation [26,36]. Both human MRC5 and avian DT40 cells lacking PrimPol (PrimPol^−/−^) also accumulate an increased number of stalled forks, or a reduced ability to restart stalled forks, following UV damage [26,28,36,41]. Unlike human cells, DT40 cells are hypersensitive to UV irradiation in the absence of PrimPol only, potentially due to the faster doubling times and increased S-phase population of these cells [26]. Interestingly, it has recently been shown that PrimPol^−/−^ avian cells are even more sensitive to UV damage than previously appreciated [65]. In fact, these cells were found to be more sensitive than those lacking Pol η when analysed by colony formation assays. This effect was determined to be due to an extended G2 arrest, which prevented cell cycle progression, rather than an increase in apoptosis [65]. These reports clearly implicate PrimPol in the maintenance of replisome progression, or restart of stalled replication forks, in the presence of UV damage lesions. 

However, PrimPol is also involved in the tolerance of other types of DNA damage. PrimPol^−/−^ DT40 cells are hypersensitivity to methylmethane sulfonate (MMS), cisplatin, and hydroxyurea (HU) [66]. Further deletion of Pols ζ and η in these cells leads to an additional increase in damage sensitivity to a similar extent as in wild-type cells, again indicating an independent role for PrimPol in DNA damage tolerance [66]. PrimPol is also required for recovery of stalled replication forks following HU treatment in HeLa cells [28,36]. Notably, each of these DNA damaging agents acts to stall the progression of replication forks. MMS causes the generation of abasic sites in the template strand, cisplatin crosslinks DNA, and HU acts to inhibit ribonucleotide reductase and, consequently, dNTP production. In contrast, loss of PrimPol does not sensitise cells to ICRF193, camptothecin, or γ-rays, agents that produce DNA strand breaks. This is suggestive of a broad role for the enzyme in damage tolerance, but not in break repair [66]. PrimPol associates with chromatin during G1 and S-phase and PrimPol^−/−^ mouse embryonic fibroblasts (MEFs) present chromosome aberrations indicative of S-phase defects, which are enhanced after aphidicolin treatment [26,36]. Collectively, these findings place PrimPol at the replication fork during S-phase and indicate a role in the tolerance of replicase-stalling DNA damage.

### 4.2. PrimPol Reprimes and Restarts Stalled Replication Forks

The DNA damage tolerance defects observed in the absence of PrimPol potentially indicates that it acts as both a TLS polymerase and a repriming enzyme. However, more recent reports clearly support the latter function [34,36,66,67,68]. Although PrimPol is described as a TLS Pol, the spectrum of DNA damage types it can traverse by ‘true’ TLS is actually rather limited. Discounting pseudo-TLS bypass, which may or may not be relevant in vivo, PrimPol is essentially only able to directly bypass 8oxoG lesions [26,27,34,36]. Moreover, a number of other Pols are also able to efficiently and accurately bypass these lesions [69,70,71]. If PrimPol’s primary role were as a TLS Pol, this observation would be at odds with the range of replicase-stalling DNA damaging agents it is involved in tolerating [66]. This implies that PrimPol most likely acts as a repriming enzyme for the tolerance of DNA damage and this is supported by the study of separation of function mutants [34,36,66,67].

Mutation of PrimPol’s ZnF domain abolishes primase, but actually enhances polymerase activity [34,36]. This important observation has permitted investigation into the requirement of primase activity for the enzyme’s role during DNA replication in vivo. In each case, when PrimPol^−/−^ cells were complemented with the primase-deficient/polymerase-proficient ZnF mutant, it was unable to rescue any of the observed damage tolerance defects [34,36,66]. In contrast, complementation with a primase-proficient/reduced-polymerase mutant of PrimPol restored DNA damage tolerance to wild-type levels [66,72]. In agreement with this, PrimPol was able to facilitate close-coupled repriming downstream of lesions in vitro, which it cannot bypass by TLS [66]. Aside from increased sensitivity to DNA damaging agents and decreased replication fork rates, PrimPol^−/−^ and knockdown cells exhibit persistent RPA foci and increased phosphorylation of Chk1 [28,36]. Both of these stress response markers are indicative of the generation of stretches of ssDNA [11,73]. This would be an expected consequence of a lack of repriming by PrimPol, resulting in the uncoupling of leading/lagging strand replication and excessive strand-specific unwinding by MCM [11]. In agreement, cells compensate for the loss of PrimPol by increasing both homologous recombination (HR) mediated fork rescue and dormant origin firing [26,33,36]. These compensatory back-up mechanisms, in addition to redundancy between PrimPol and TLS polymerases, may explain why PrimPol is dispensable for viability in human cells and mouse models [27]. These observations give further credibility to a requirement for PrimPol at the progressing replication fork during S-phase, which might not necessarily be the case if a TLS-like role was being performed. 

As previously mentioned, TLS can potentially occur both at the replication fork, as well as post-replicatively, to fill in gaps left opposite lesions following repriming or dormant origin firing [16]. Each of these possibilities are not mutually exclusive, but a number of studies point to post-replicative gap-filling as the predominant role for TLS. In yeast, DNA damage tolerance mechanisms, including TLS, have been found to operate effectively in a post-replicative manner, and ssDNA gaps, indicative of repriming, accumulate following UV-damage [11,17,18]. Likewise, in human cells DNA replication fork progression in the presence of UV damage was found to be independent of TLS and ssDNA gaps opposite UV lesions were identified. It was concluded that these gaps were likely a result of repriming downstream of lesions rather than dormant origin firing [12]. Importantly, mutation of Pol η or other TLS factors does not appear to significantly alter replication fork rates in the presence of damage [12,20]. This is in stark contrast to the effect of loss of PrimPol on replication fork progression following damage, further supporting a repriming, rather than TLS, role for this enzyme in vivo. 

### 4.3. PrimPol Bypasses Non-Canonical Replication Impediments

Whilst DNA damage lesions are some of the best characterised replication impediments, they are not the only obstacles replication forks must overcome during their progression. In addition to the B-form of dsDNA we have become familiar with since Watson and Crick’s famous model [74], genomic DNA can also adopt a number of other secondary structures as a result of specific sequence motifs and protein interactions [75]. One alternative DNA secondary structure, which has received increasing attention as evidence for its formation in vivo grows, is the G-quadruplex (G4) [76]. G4s are produced by the stacking of G-quartets, which form through alternative Hoogsten base-pairing between guanine bases. These structures may potentially play an important role in transcription and DNA replication in the cell, but they can also pose as major impediments to replisome progression [77,78,79,80]. Consequently, cells possess a number of specialised helicases and Pols to replicate past G4s [81]. 

Previously, cells lacking fanconi anemia complementation group J (FANCJ) or REV1 DNA-directed polymerase were found to stochastically lose Bu-1a protein expression [82,83]. Importantly, the *BU-1A* locus contains a G4, which was determined to stall replication in these cells. This stalling causes uncoupling of replication from histone recycling at the *BU-1A* locus and consequently leads to the deletion of epigenetic marks, manifesting in loss of Bu-1a expression. It was recently identified using Bu-1a read-out assays that PrimPol also plays a critical role in the bypass of these structures during DNA replication [67]. Consistent with PrimPol’s behaviour at most DNA damage lesions, in vitro analysis revealed that the enzyme is unable to directly read through G4s, but can bind to and facilitate close-coupled repriming downstream of these structures. Vitally, close-coupled repriming ≈6 nt ahead of the G4 would permit the appropriate recycling of histones, and thus maintain epigenetic marks and Bu-1a expression. Bypass of G4 structures through repriming by PrimPol was confirmed in vivo using the ZnF primase-deficient mutant discussed previously. Here, complementation of PrimPol^−/−^ cells with the ZnF mutant failed to prevent instability of Bu-1a expression, in contrast to the wild-type protein, confirming that PrimPol’s primase activity is critical for G4 bypass [67]. Intriguingly, PrimPol was found to only be required for G4 bypass during leading strand replication. Presumably, this is because primers are constantly generated on the lagging strand due to the discontinuous nature of DNA synthesis on this strand. 

Further evidence supporting a general role for PrimPol in repriming replication downstream of fork-stalling obstacles is provided by studies of chain-terminating nucleoside analogues (CTNAs) [66]. CTNAs cause replication to stall, when incorporated into the 3’-termini of growing DNA polymers, by preventing further extension as they lack the 3’ hydroxyl required for phosphodiester bond formation [84,85]. Loss of PrimPol has been shown to cause hypersensitivity to a wide range of CTNAs [66]. Critically, the inability of Pols to extend from CTNAs rules out bypass by direct extension. PrimPol was found to be important for the tolerance of CTNAs by repriming downstream. This role was confirmed by both in vivo characterisation of the ZnF mutant and in vitro analysis of repriming synthesis after CTNAs [66]. 

These critical findings not only establish that PrimPol deploys a repriming mechanism to bypass G4s and CTNAs, in a similar manner to DNA damage lesions, they also point to the possibility that PrimPol is able to bypass a wide range of leading strand obstacles during normal and perturbed replication. This is in contrast to canonical TLS Pols, which are typically highly specialised in the lesions they can bypass. Consequently, it is likely PrimPol is broadly employed as a general mechanism to reprime and restart replication ahead of many different leading strand replication impediments. 

### 4.4. A Role for PrimPol in Mitochondrial DNA Replication?

The majority of genetic information in mammalian cells is stored in the nucleus. However, a small proportion of DNA is also located in the mitochondria. Despite being only ≈16.6 kb long and encoding just 13 polypeptides, mutation of the mitochondrial genome is responsible for a number of mitochondriopathies and is implicated in various other pathologies including cancer, cardiovascular diseases, and neurodegenerative disorders [86]. Unlike nuclear DNA, cells possess many copies of mtDNA making it highly redundant. In line with this, the rate of mutagenesis is ≈10-fold greater in the mitochondria than the nucleus [86]. A major function of mitochondria is the generation of ATP through oxidative phosphorylation (OXPHOS). This process produces reactive oxygen species (ROS) which can induce damage lesions, including 8oxoG and Ap sites, in mtDNA [86].

A significant proportion of PrimPol has been found to localise to the mitochondria where it interacts with mitochondrial single-strand binding proteins (mtSSB), suggesting a potential role in the tolerance of mtDNA damage [26,27,55,87]. This is supported by defects in mtDNA replication and copy number observed in cells lacking PrimPol [27,87]. However, the ability to generate viable PrimPol^−/−^ mice demonstrates that this role is redundant. Indeed, mitochondrial RNA Pol (POLRMT) is likely responsible for generating the initial primers essential for mtDNA replication [88]. These primers are then extended by Pol γ, which until recently was thought to be the only mitochondrial DNA Pol [89]. In addition to PrimPol, more recent reports indicate that Pol θ and Pol ζ are also involved in human mtDNA replication [90,91].

Given that few TLS Pols appear to localise to the mitochondria, in addition to the high levels of ROS there, it was speculated that PrimPol may be involved in TLS bypass of mitochondrial 8oxoG lesions and Ap sites [27]. In order to investigate this, a recent study analysed the ability of PrimPol to assist the mitochondrial replisome in oxidative damage bypass by TLS [42]. Here, it was found that the mitochondrial replisome is completely stalled by Ap sites and pauses significantly at 8oxoG lesions. PrimPol did not enhance the bypass of either of these lesions, disagreeing with a TLS role in oxidative damage bypass in the mitochondria [42]. Thus, it seems more likely that PrimPol functions to reprime mtDNA replication downstream of blocking lesions, similar to its role in the nucleus. In addition to oxidative damage, mtDNA is also subject to deletions. Intriguingly, these deletions map in close proximity to G4-forming sequences [92]. In light of the role of PrimPol in repriming after G4s in nuclear DNA replication, it would not be surprising if the enzyme fulfilled the same role in the mitochondria. However, further work is required to confirm a repriming role for PrimPol here. The potential role for PrimPol in the mitochondria has recently been reviewed in more detail [93].

### 4.5. Is PrimPol Involved in Somatic Hypermutation?

Generally, mutagenesis during DNA replication is avoided at all costs in order to preserve genomic stability. However, an exception to this is during the development of the immune system. Here, mutagenesis occurs in immunoglobulin (Ig) genes to enable variation in the generated antibodies. This programmed mutagenesis is driven by activation-induced deaminase (AID), which deaminates dC to dU [94]. Replication of dU facilitates C>T transitions. Additionally, dU may be further processed by uracil DNA glycosylase (UNG) to generate Ap sites. TLS bypass of these Ap sites can alternatively create C>A/G/T mutations due to the non-instructive nature of the lesion [95]. 

The involvement of TLS Pols in somatic hypermutation (SHM) at Ap sites led to speculation that, if PrimPol functions as a TLS Pol in vivo, it might also modulate this mutagenesis. Analysis of DT40 cells found that hypermutation and gene-conversion events are similar in wild-type and PrimPol^−/−^ cells [66]. Moreover, loss of PrimPol in wild-type and Pol η^−/−^/Pol ζ^−/−^ avian cells did not significantly alter the mutation spectrum of the studied Ig gene. Intriguingly, another report, which analysed large mutational data sets in mice, identified that PrimPol does have a subtle effect on SHM outcome [68]. In this study, loss of PrimPol was found to selectively increase C>G transversions, but did not affect other G/C or A/T mutations. Interestingly, PrimPol was found to specifically prevent the generation of C>G transversions in the leading strand, potentially explaining the G>C over C>G strand bias of somatically mutated IgH loci [68]. However, this anti-mutagenic activity of PrimPol was attributed to the enzyme’s primase, rather than TLS polymerase, activity. It was concluded that PrimPol preferentially reprimes downstream of Ap sites on the leading strand, therefore maintaining fork progression and preventing error-prone TLS. The resulting ssDNA gap opposite the Ap site could then be filled in by error-free homology directed repair. Fascinatingly, in the same report, studies of invasive breast cancers suggested that this leading strand anti-mutagenic activity of PrimPol may be genome wide. 

Together, these reports establish that PrimPol does not act as a canonical TLS polymerase during SHM. Rather, PrimPol affects the mutational outcome of SHM by repriming downstream of Ap sites on the leading strand thus preventing C>G transversions. These findings, therefore, further support mounting evidence that PrimPol’s primary role in DNA damage tolerance is to reprime leading strand replication and not to perform TLS. 

### 4.6. Why Doesn’t the Pol α-Primase Complex Reprime Leading Strand Replication?

The emerging role for PrimPol in repriming leading strand replication begs the question; why doesn’t the replicative Pol α-primase complex fulfil this role? In *E. coli*, DnaG, the replicative primase, efficiently reprimes replication ahead of replicase stalling DNA damage lesions, permitting bypass of the damage without dissociation of the replisome [13,14]. Likewise in yeast which lack PrimPol, leading strand repriming is presumably facilitated by Pol α-primase, suggesting that, at least in these organisms, the replicative primase has the capacity to also fulfil this role. 

Whilst the answer to this question is not completely clear, PrimPol does have one advantage over Pol α-primase; it preferentially primes using dNTPs. This minimises the amount of RNA processing required on the leading strand. Although ribonucleotides are routinely incorporated during the initiation of each Okazaki fragment on the lagging strand and at replication origins on the leading strand, their persistent presence in DNA can lead to genomic instability [96]. Ribonucleotides incorporated during primer synthesis are routinely removed through Okazaki fragment maturation [97]. However, it is not clear how a DNA secondary structure or lesion requiring bypass upstream of the primer would affect this process. 

Ribonucleotides incorporated by replicative Pols are removed by ribonucleotide excision repair (RER). Intriguingly, in RER deficient yeast leading strand ribonucleotides are removed through a topoisomerase I (Top1) mediated mechanism, which likely also removes a subset of ribonucleotides in RER proficient cells [97,98]. This mechanism of ribonucleotide removal, which does not appear to occur on the lagging strand, is susceptible to causing genome instability. This makes ribonucleotides present in the leading strand potentially more detrimental than those in the lagging strand. This is supported by observations that loss of RER and increased ribonucleotide incorporation by Pol ε, but not Pol α or Pol δ, is lethal [98].

Although RER deficient yeast are viable, loss of this pathway in mice results in embryonic lethality [99]. Thus, the greater pressure on higher eukaryotes to minimise the presence of ribonucleotides in their genomes may explain why PrimPol is employed for leading strand repriming using dNTPs in these organisms. However, this enzyme has been lost in some lower eukaryotes as an alternative repriming mechanism, possibly involving the replicative primase, appears to be available. 

### 4.7. Why Is PrimPol Damage Tolerant In Vitro?

If PrimPol’s primary role in vivo is to reprime DNA replication, why does the enzyme display TLS-like activity in vitro? Although it is possible that PrimPol’s TLS-like activity is important in the cell, recent studies suggest that the enzyme’s primase activity is more relevant for its in vivo role, as discussed above. This opens up the possibility that this TLS activity is a ‘side effect’ of being a primase and this is supported by a number of observations. 

Recent studies of the RNA primase domains of human Pol α-primase provide insight into the unique way primases interact with their DNA template and primer [100,101]. The RNA primase associated with Pol α is a heterodimer composed of a small catalytic subunit, p49, and a large regulatory subunit, p58. These reports identify that the C-terminal domain of p58 binds to the DNA/RNA junction at the 5’-end of the RNA primer, whereas p49 binds and extends the 3’ end of the primer moving away from p58. The p49 subunit makes few contacts with the DNA/RNA, resulting in distributive activity. By only contacting the primer at the 5’ and 3’ ends, the primase is unable to sense modified nucleotides in the RNA strand, potentially explaining the propensity of primases to perform TLS-like extension [100,101]. The authors suggest that this binding mechanism is broadly applicable to most primases. In the context of PrimPol, the ZnF is likely functionally equivalent of p58. Indeed, both are flexibly tethered to the catalytic domain and required for template recognition during priming, although PrimPol’s ZnF has only been shown to bind ssDNA [34,102]. Nevertheless, the ZnF domain may bind the ssDNA immediately upstream of the 5’ end of the primer. 

The crystal structure of PrimPol’s AEP domain potentially supports this model [35]. Here, only the templating base is held in the active site cleft, with the rest of the 5’ template strand directed out of the catalytic centre. Additionally, PrimPol lacks a thumb domain and makes few contacts with the primer strand. This potentially prevents the enzyme from sensing damaged bases in the template and allows them to be looped out. Furthermore, unlike TLS Pols, PrimPol does not possess an ‘open’ active-site cleft and is unable to accommodate bulky lesions such as CPDs and 6-4PPs [35]. This provides further evidence that PrimPol is not a ‘true’ TLS Pol, rather it loops out bulky-lesions during bypass, resulting in deletions. 

The ability of primase-polymerases to perform TLS-like extension is well documented [24]. Some AEPs have co-opted this inherent catalytic versatility for use in other processes such as NHEJ, becoming specialised and in some instances, losing their ability to prime [24]. However, PrimPol’s primase activity is critical for its role in vivo and thus it is possible that the TLS-like activities observed in vitro simply arise as a by-product of the structural features necessary for priming. 

## 5. How Does PrimPol Get to Where It is Needed? The Recruitment of PrimPol to Stalled Replication Forks

The studies described above strongly indicate that PrimPol’s main role in DNA replication is to reprime ahead of impediments on the leading strand. In order to fulfil this role, PrimPol must be efficiently recruited to ssDNA downstream of stalled replication forks. In this section, we will describe recent advances in our understanding of the interactions and mechanisms governing recruitment of PrimPol. 

### 5.1. PrimPol Interacts with Single-Strand Binding Proteins

Replication fork stalling can cause uncoupling of leading and lagging strand synthesis, consequently generating ssDNA stretches on either strand due to continued unwinding by the replicative helicase [11]. The impact of this on the lagging stand is likely limited by the generation of new Okazaki fragments. However, in the absence of leading strand fork restart, extended uncoupling can produce stretches of ssDNA. In nuclear DNA replication, the resulting ssDNA is bound by RPA, which in turn can trigger the S phase checkpoint response [103]. 

Unlike TLS Pols, PrimPol does not interact with proliferating cell nuclear antigen (PCNA) [55]. However, it does interact with both the major nuclear and mitochondrial single-strand binding proteins (SSBs); RPA and mtSSB [28,55]. PrimPol’s interaction with RPA is mediated by its C-terminal domain (CTD), which binds to the N-terminus of RPA70 (RPA70N), the largest subunit of the RPA heterotrimer [55]. The structural basis for PrimPol’s interaction with RPA has recently been elucidated [104], identifying that PrimPol possesses two RPA binding motifs (RBMs) in its CTD (RBM-A and RBM-B), which both bind to the basic cleft of RPA70N, independently of each other. Interestingly, this cleft has previously been shown to interact with, and recruit, a number of different DNA damage response proteins, including RAD9, MRE11, ATRIP, and p53 [105]. 

Together, these studies indicate that PrimPol may also be recruited to stalled replication forks through its interaction with RPA; with mtSSB likely playing an analogous role in mitochondria. 

### 5.2. RPA Recruits PrimPol to Stalled Replication Forks

Previously, it was identified that PrimPol’s CTD is required for its function and co-localisation with RPA in vivo [28]. However, interpretation of these results is limited as removal of the whole CTD has been shown to reduce primase activity in vitro and may also abrogate interactions with other binding partners [34]. Structural studies of PrimPol-RPA complexes have enabled the in vivo analysis of point mutants that disrupt this interaction. These studies identified that PrimPol’s RBM-A is the primary mediator of the RPA interaction in vivo, whilst RBM-B appears to play a secondary role. Furthermore, RBM-A mutants were unable to restore replication fork rates following UV-damage, in comparison to the wild-type or RBM-B mutant protein [104]. These findings revealed that PrimPol’s interaction with RPA is required for its cellular role. Moreover, this study also showed that this interaction is responsible for the recruitment of PrimPol to chromatin, demonstrating that the enzyme is recruited to stalled replication forks by RPA [104]. Intriguingly, mutations of key residues in each RBM have been identified in cancer patient cell lines, adding further support that these motifs are important for PrimPol’s function in vivo [104]. 

Aside, from identifying the mechanism by which PrimPol is recruited to stalled replication forks; these studies also add to the growing evidence supporting a role for PrimPol as a repriming enzyme. PrimPol’s recruitment to RPA, and lack of interaction with PCNA, suggests it binds to ssDNA downstream of a stalled replicase on the leading strand, the ideal place to facilitate repriming following initial leading/lagging strand uncoupling to prevent excessive ssDNA generation. A recent report investigating the role of RAD51 recombinase (RAD51) in aiding replication across UV lesions supports this [106]. Here, RAD51 and MRE11 depletion was found to favour ssDNA accumulation at replication obstacles and subsequent PrimPol-dependent repriming. This also supports previous suggestions that excessive unwinding of DNA following stalling of the replicase is sufficient to promote ssDNA generation and repriming at replication impediments [12]. 

Further work is required to elucidate the exact mechanisms controlling PrimPol’s recruitment by RPA to ssDNA. Interestingly, binding of MRE11 and RAD9 to RPA is enhanced upon RPA32C phosphorylation [107,108]. Thus, phosphorylation of RPA may act to signal recruitment of DNA damage response proteins, potentially including PrimPol [109].

## 6. Regulation of PrimPol during DNA Replication

**Figure 3:**
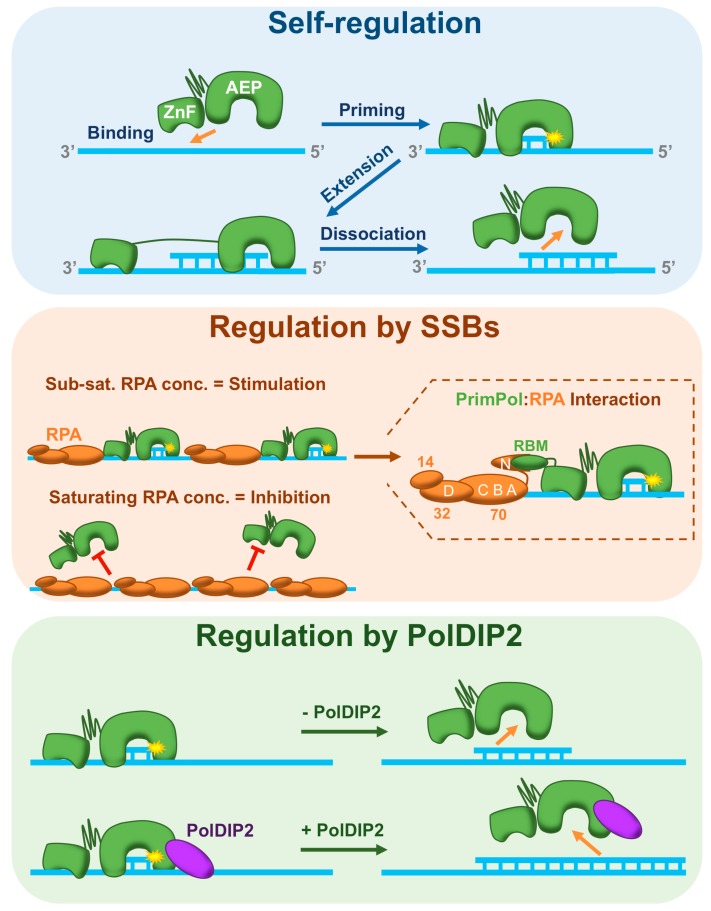
Regulation of PrimPol by its ZnF domain and interacting partners. **Top panel**: PrimPol is inherently self-regulatory due to the restraining effect of its ZnF domain. The AEP and ZnF domains of PrimPol form a hinge-like structure, connected by a flexible linker. Binding of PrimPol to ssDNA is mediated by the ZnF domain, which binds 3’ relative to the AEP domain on the template strand. Binding of the ZnF stabilises the AEP domain, permitting primer synthesis. The AEP then extends the primer, but is restricted by the maximum distance it can move away from the ZnF. The enzyme subsequently dissociates leaving behind a short primer. This mechanism limits the processivity of the PrimPol; **Middle panel:** PrimPol is regulated by single-strand binding proteins (SSBs). At sub-saturating concentrations of RPA, the protein acts to recruit PrimPol to the ssDNA template, consequently stimulating primer synthesis. In vivo, this interaction is primarily mediated by PrimPol’s RBM-A, which binds to the basic cleft of RPA70N. At saturating RPA concentrations, when the ssDNA template is fully coated, PrimPol cannot gain access and primer synthesis is inhibited. This serves to limit where PrimPol can prime; **Bottom panel:** Polymerase-delta interacting protein 2 **(**PolDIP2 or PDIP38) enhances PrimPol’s primer extension activity by binding the AEP domain and stabilising it on DNA.

Recent reports strongly indicate that PrimPol is recruited by RPA to the leading strand, following replicase stalling, in order to reprime replication and prevent genome instability. However, PrimPol is an error-prone enzyme and unscheduled or dysregulated activity could lead to mutagenesis [55]. In this section, we will discuss our current understanding of the mechanisms used to limit PrimPol’s contribution to DNA synthesis during replication (Figure 3). 

### 6.1. Regulation of the Cellular Concentration of PrimPol

The simplest way to regulate the activity of a protein is by controlling its intracellular concentration. This is especially true for proteins that are only required to act in response to a specific stress, for example DNA damage response proteins. This strategy is utilised during the SOS response in *E. coli*. Here, ≈40 DNA damage response genes are upregulated in response to DNA damage [110]. 

In comparison to Prim1, PrimPol is expressed at very low levels in human U2OS cells (<500 protein copies per cell compared to ≈13,300) [111]. This is, however, similar to the expression level of TLS Pols, including η and κ. PrimPol mRNA expression peaks in G1-S phase, although the total protein levels remain roughly constant throughout the cell cycle [36]. Thus, the increased association of PrimPol with chromatin during the G1 and S phases of the cell cycle in unperturbed cells is a result of finer mechanisms controlling recruitment to DNA, rather than increased expression. This may also be the case with the increased recruitment of the enzyme to chromatin in response to DNA damage. Nevertheless, the low level of PrimPol expression, in comparison to the replicative primase, acts as the primary mechanism to restrict its contribution to ‘normal’ replication. 

### 6.2. PrimPol Is Self-Regulating

The structural features afforded to PrimPol by virtue of being a primase also act as inherent regulatory mechanisms. As mentioned previously, PrimPol displays very low processivity. This distributive nature appears to be due to two key features. Firstly, the AEP catalytic domain has a much smaller ‘footprint’ than most polymerases, potentially explaining why the enzyme binds so poorly to DNA [35]. Secondly, the ZnF domain acts to negatively regulate PrimPol’s processivity (Figure 3, top panel) [34]. 

It has been suggested that the p58 subunit of the replicative eukaryotic primase enforces a strict counting mechanism on the enzyme [112]. Here, the p58 and p49 subunits form a hinge-like structure. The enzyme binds to ssDNA in a ‘closed’ conformation, with p58 facilitating template recognition. The p49 subunit then initiates primer synthesis, moving away from p58 that binds the 5’ end of the primer [101,112]. Thus, an inherent counting mechanism is conferred by the maximum distance p49 can elongate the primer strand away from p58. The ZnF domain of PrimPol is thought to act in a similar way [104]. In this scenario, the AEP domain and ZnF may form a hinge-like structure, connected by a flexible linker. The enzyme probably binds to DNA in a closed conformation assisted by the ZnF domain, which binds on the 3’ side relative to the AEP domain on the template strand. The AEP domain can then synthesise and elongate the primer strand until further extension is restricted by the ZnF domain (Figure 3, top panel). It is also conceivable that the AEP and ZnF domains bind DNA in an open conformation, with the ZnF bound on the 5’ side relative to the AEP on the template strand, extension would then be limited by inter-domain collisions. In the absence of the ZnF, PrimPol displays increased, but still poor, processivity due to the weak affinity of the AEP domain for the DNA template [34]. 

PrimPol is, therefore, self-regulating. The supervisory effect of the ZnF domain, which permits priming but limits elongation, coupled with the AEP’s poor affinity for DNA, restricts the ability of PrimPol to partake in significant unregulated DNA synthesis during DNA replication.

### 6.3. Regulation by Single-Strand Binding Proteins

The ability of primases to bind and prime on ssDNA gives them the potential to facilitate unscheduled priming in vivo, wherever ssDNA is available. Despite limiting the synthesis of long DNA tracts, PrimPol’s self-regulatory mechanisms do not restrict where it can prime. Dysregulated priming is potentially highly detrimental to the cell, as these primers could be extended by other Pols. To prevent this, PrimPol is also regulated by RPA and mtSSB (Figure 3, middle panel). Both of these SSBs stimulate the activity of their respective replicative Pols, δ and γ [113,114]. In contrast, both RPA and mtSSB severely restrict the polymerase activity of PrimPol [55]. Additionally, these SSBs can also inhibit primase activity, as is the case with Pol α-primase [55,115]. More recently, it was reported that RPA’s effect on PrimPol’s primase activity is highly concentration-dependent. In fact, sub-saturating concentrations of RPA dramatically stimulate primer synthesis but inhibition occurs as the concentration increases [104]. 

It is likely that both RPA and mtSSB act to prevent unscheduled priming events by blocking access to the DNA template. Thus, PrimPol requires a free ssDNA interface adjacent to the SSB in order to be recruited (Figure 3, middle panel). This recruitment likely acts to enhance PrimPol’s poor affinity for DNA, providing a platform for primer synthesis. 

RPA binds ssDNA with a defined polarity [116,117,118,119]. Initially, the DNA-binding domain A (DBD-A) and DBD-B oligonucleotide binding (OB) folds of RPA70 bind ssDNA in a tandem manner, forming an 8-nt binding complex. The interface in contact with DNA is then extended to 20–30 nts by the binding of DBD-C and DBD-D, which occurs in a defined 5’-3’ direction on the template strand [120]. This would likely position the RPA70N domain, which recruits PrimPol, 5’ relative to rest of the RPA molecule on the template strand (Figure 3, middle panel). This suggests that PrimPol binds ahead of RPA in vivo, with the ZnF contacting ssDNA adjacent to RPA and the AEP bound downstream. 

The orientation of PrimPol’s interaction with RPA may explain the inhibition observed in primer extension assays. By preferentially binding on the 5’ side of RPA, PrimPol would not be able to access the primer stand at the 3’ end of the template. Additionally, replicative Pols are thought to be able to easily displace RPA as they approach the protein from the 3’ side, encountering the weakly bound DBD-D and DBD-C domains, before DBD-B and DBD-A [121]. This in turn shifts the equilibrium from the 20–30-nt RPA complex, to the more weakly bound 8-nt mode, thus permitting displacement. In contrast, if PrimPol binds to the 5’ side of RPA, it would move away from the protein, preventing displacement in the same way. It is likely that this interaction also further enhances the regulation of PrimPol’s processivity by ‘holding’ the ZnF domain and preventing continued extension by the AEP domain. 

### 6.4. What Generates the ssDNA Interface Required for PrimPol Recruitment?

The requirement of a ssDNA interface downstream of RPA for efficient PrimPol recruitment begs the question: how is this free ssDNA interface generated in vivo? Although the answer to this question is currently unknown, one obvious solution would be through the action of the replicative helicase. Following stalling of the leading strand replicase, leading and lagging strand replication can become uncoupled. Here, the replisome progresses in the absence of DNA synthesis on the leading strand. Continued unwinding of duplex parental DNA by MCM generates ssDNA on the leading strand, which is bound by RPA. Consequently, an RPA/ssDNA interface for PrimPol binding could be generated directly behind the progressing MCM. Subsequent repriming by PrimPol would prevent extended leading/lagging strand uncoupling, allowing leading strand replication to resume at the progressing replisome. The short RPA-bound ssDNA gap left behind could then be filled by TLS or template switching mechanisms. 

In support of this, it has recently been shown that the mitochondrial replicative helicase, Twinkle, can stimulate DNA synthesis by PrimPol, indicating that replicative helicases can potentially facilitate PrimPol activity in vivo [42]. It is interesting to note that many DNA primases interact with replicative helicases, with some even possessing their own helicase domains [112].

### 6.5. Regulation by PolDIP2

PolDIP2 was originally identified as a binding partner of the p50 subunit of Pol δ, in addition to PCNA [122]. More recently, PolDIP2 was shown to interact with Pols η, ζ, λ, and Rev1 [70,123]. in vitro, the protein stimulates the polymerase activity of Pol δ by increasing its affinity for PCNA, as well as enhancing TLS by Pols η and λ [70]. These observations have led to suggestions that PolDIP2 may play an important role in the switch between Pol δ and TLS polymerases during DNA replication [70,123]. 

**Figure 4:**
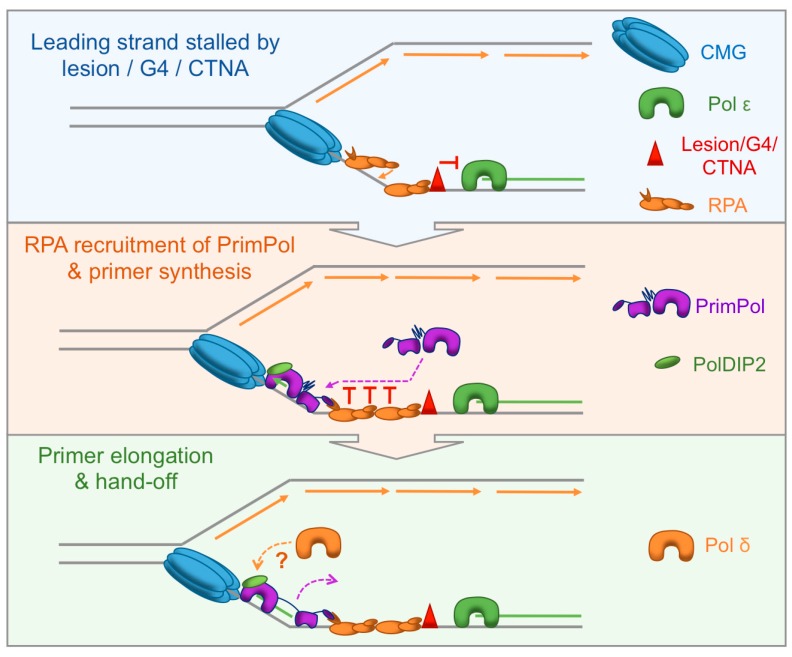
Role, recruitment, and regulation of PrimPol during DNA replication. **Top panel:** Pol ε is stalled on the leading strand by a lesion, secondary structure, or CTNA. Lagging strand replication continues, subsequently generating ssDNA on the leading strand. This ssDNA is bound by RPA as the CMG complex progresses; **Middle panel:** The generation of an RPA / ssDNA interface provides a platform for PrimPol recruitment. PrimPol requires a free ssDNA region adjacent to RPA and thus is recruited to the exposed ssDNA behind the CMG complex. This recruitment is facilitated by the interaction between PrimPol’s RBMs and RPA70N. Following recruitment, PrimPol reprimes the leading strand; **Bottom panel:** PrimPol elongates its primer, assisted by PolDIP2, before further extension is restricted by its ZnF and RPA interaction. The primer is then handed-off to the replicative polymerase, possibly Pol δ, mediated by each protein’s interaction with PolDIP2.

PolDIP2 also significantly enhances the DNA binding and processivity of PrimPol’s AEP domain (Figure 3, bottom panel) [41]. Additionally, PolDIP2 appears to be important for PrimPol’s function in vivo, suggesting it may act as a way to positively regulate the enzyme’s activity. Notably, however, this was not sufficient to relieve the negative effect of RPA or mtSSB on PrimPol’s polymerase activity. It seems likely that PolDIP2 acts to assist PrimPol’s AEP domain during primer extension after synthesis of the initial di-nucleotide, without necessarily allowing synthesis of long DNA tracts. Interestingly, PolDIP2 binds to PrimPol at a region in close proximity to motifs Ia and Ib, identified in the recent crystal structure of PrimPol [35,41]. These motifs harbour the majority of the residues responsible for mediating binding of the AEP domain to the DNA template. PolDIP2, therefore, potentially changes the conformation of this region to enhance PrimPol’s affinity for the DNA template, resulting in increased DNA binding and processivity. Additionally, PolDIP2 may also serve as a hand-off mechanism to the replicative Pol, following primer synthesis by PrimPol (Figure 4). 

Intriguingly, Pol δ has recently been implicated in extension of a small fraction of primers synthesised by Pol α-primase on the leading strand during DNA replication in yeast [124]. Given that yeast lack PrimPol, this small fraction of primers could in theory be products of repriming by Pol α-primase. This raises the fascinating possibility that Pol δ can serve to extend primers on the leading strand following a repriming event, before subsequent replacement by Pol ε. This could possibly be due to the stalling of Pol ε at the initial impediment, or alteration of the core replisome following leading/lagging strand uncoupling. In higher eukaryotes, these leading strand repriming events appear to be facilitated by PrimPol, not Pol α-primase. Thus, PolDIP2 may act as a hand-off mechanism from PrimPol to Pol δ, given the interaction with both proteins and ability of PolDIP2 to enhance the Pol δ/PCNA interaction. However, more work is required to investigate this possible mechanism (Figure 4). 

## 7. Conclusions and Perspectives

Nearly half a century ago, Rupp and Howard-Flanders identified the presence of ssDNA gaps left opposite UV photoproducts following DNA replication in nucleotide excision repair deficient *E. coli* [125]. A model was proposed which envisaged re-initiation of replication downstream of the damage on both leading and lagging strand templates; the first suggestion of repriming. The idea of leading strand re-initiation remained controversial until almost four decades later when origin-independent leading strand re-initiation was observed [126]. Follow-up studies confirmed that the replicative primase, DnaG, could reprime leading strand replication downstream of a lesion, whilst the replisome remained associated with the template [13,14]. Over recent years, evidence has accumulated to support leading strand repriming as a conserved mechanism for dealing with replisome-stalling impediments in eukaryotes [11,12,18] and recent studies have established that PrimPol’s major role in eukaryotic organisms is to act as a primase that facilitates the bypass of a wide range of leading strand obstacles (Figure 4) [34,36,66,67,68,104,106].

Since the initial reports describing PrimPol only three years ago, studies from a number of laboratories have greatly increased our understanding of the role, recruitment, and regulation of the enzyme during DNA replication [26,27,28]. However, we are only just beginning to appreciate the novel roles that PrimPol plays in DNA replication and damage tolerance. The exact interplay between leading strand repriming by PrimPol and other DNA damage tolerance mechanisms, such as TLS, is still not yet clear. It is possible that DNA damage tolerance mechanisms work to complement repriming by filling in the resulting ssDNA gaps. Alternatively, repriming could occur when TLS at the replication fork fails, in order to prevent extended leading/lagging strand replication uncoupling. The redundancy between Pol α-primase and PrimPol in vivo is also an interesting avenue for future studies. The reason for the apparent requirement of PrimPol for leading strand repriming in higher eukaryotes, but not other organisms, is not yet completely clear. Although leading strand repriming is emerging as the primary role for PrimPol during DNA replication, the catalytic versatility of the enzyme may lend itself to disparate roles in other processes, such as transcription [43]. 

We now know that RPA serves to recruit PrimPol to stalled replication forks in the nucleus [104]. However, mtSSB has not yet been shown to play an analogous role in the mitochondria, although an interaction between these proteins in vivo has been reported [55]. Additionally, it is possible that post-translational modifications, as well as interactions with the replicative helicases, play a role in this process [42]. The necessity of appropriate recruitment and regulation of PrimPol in the cell is highlighted by the mutations of PrimPol’s RBMs identified in cancer patient cell lines, which likely adversely affect recruitment of the enzyme [104]. The regulation of PrimPol appears to walk a fine line between preventing and causing genetic instability, as PrimPol is inherently error-prone and also been found to be over-expressed in some cancers, such as glioma [55,127]. Although we have highlighted some of the known mechanisms regulating PrimPol’s activity here, it is likely that additional layers of regulation remain to be discovered. 

The hypersensitivity to DNA damaging agents observed in absence of PrimPol legitimises the enzyme as a potential target for inhibition in combination with other DNA damage tolerance factors and DNA damaging chemotherapeutics [26,36,66]. Similarly, PrimPol homologues in trypanosomes have been identified as essential for survival and thus PrimPol-like proteins in other species may also be potential targets for anti-parasitic drugs [33]. Further studies will be important in determining the viability and usefulness of manipulating PrimPol in treating cancer and other diseases. 

## Data Statement

No new data were created during this study.
